# Does the Pollen Diet Influence the Production and Expression of Antimicrobial Peptides in Individual Honey Bees?

**DOI:** 10.3390/insects9030079

**Published:** 2018-07-04

**Authors:** Jiří Danihlík, Mária Škrabišová, René Lenobel, Marek Šebela, Eslam Omar, Marek Petřivalský, Karl Crailsheim, Robert Brodschneider

**Affiliations:** 1 Department of Protein Biochemistry and Proteomics, Centre of the Region Haná for Biotechnological and Agricultural Research, Faculty of Science, Palacký University in Olomouc, Šlechtitelů 27, 783 71 Olomouc, Czech Republic; Jiri.danihlik@upol.cz (J.D.); Rene.Lenobel@upol.cz (R.L.); Marek.Sebela@upol.cz (M.Š.); 2 Department of Biochemistry, Faculty of Science, Palacký University in Olomouc, Šlechtitelů 27, 783 71 Olomouc, Czech Republic; Marek.Petrivalsky@upol.cz; 3 Department of Molecular Biology, Centre of the Region Haná for Biotechnological and Agricultural Research, Faculty of Science, Palacký University in Olomouc, Šlechtitelů 27, 783 71 Olomouc, Czech Republic; Maria.Skrabisova@upol.cz; 4 Institute of Biology, University of Graz, Universitätsplatz 2, 8010 Graz, Austria; eslamomar@aun.edu.eg (E.O.); karl.crailsheim@uni-graz.at (K.C.)

**Keywords:** *Apis mellifera*, apidaecin, abaecin, gene expression, dietary proteins, pollen

## Abstract

We investigated the importance of protein nutrition for honey bee immunity. Different protein diets (monofloral pollen of *Helianthus* spp., *Sinapis* spp., *Asparagus* spp., *Castanea* spp., a mixture of the four different pollen and the pollen substitute Feedbee^TM^) were fed to honey bees in cages *ad libitum*. After 18 days of feeding, apidaecin 1 isoforms concentration in the thorax were measured using nanoflow liquid chromatography coupled with mass spectrometry. Expression levels of genes, coding for apidaecins and abaecin in the abdomen were determined using quantitative PCR. The results indicate that protein-containing nutrition in adult worker honey bees can trigger certain metabolic responses. Bees without dietary protein showed lower apidaecin 1 isoforms concentrations. The significantly lowest concentration of apidaecin 1 isoforms was found in the group that was fed no pollen diet when compared to *Asparagus*, *Castanea*, *Helianthus*, and *Sinapis* pollen or the pollen supplement FeedBee^TM^. Expression levels of the respective genes were also affected by the protein diets and different expression levels of these two antimicrobial peptides were found. Positive correlation between concentration and gene expression of apidaecins was found. The significance of feeding bees with different protein diets, as well as the importance of pollen nutrition for honey bee immunity is demonstrated.

## 1. Introduction

The lack of important nutrients in bee diet can result in a reduction in larval number or in a weakened vitality of adults in honey bee colonies [1]. The most needed nutrients are sugars, proteins, and lipids contained in nectar and pollen. Adult bees require an appropriate and balanced diet during their lifetime, as they are confronted with variable tasks and challenges. The nutritional quality of pollen differs among plant species, with a significant variability in the total content of proteins, lipids, sugars, and amino acids, and also in the antioxidant capacity [2]. The average pollen intake per honey bee is 3.4–4.3 mg/day, with the highest amount of pollen being consumed by nurse bees [3]. Besides the natural diet sources collected by bees during the season (nectar, honeydew, pollen), different carbohydrate diets that are fed to colonies as winter food are known to elicit differential expressions of various genes in bee fat bodies [4].

Social insects, in general, possess a lower number of immune-related genes when compared to solitary insects [5,6]. There are three levels of bee immunity that are reflected in their interactions with pathogens—physical barriers, cellular immunity, and humoral immunity, and moreover, bees as social insects evolved a special type of immunity—the social immunity [7,8]. The nutritional intake of honey bees and the effectiveness of individual immune responses appear in a possible mutual relationship [9]. The humoral part of the worker bee immunity consists of enzymes, lectins, and antimicrobial peptides (AMP). Gätschenberger et al. [10] showed that the overall immune power of summer and winter bees remained at the same level. However, when bees are highly infected with the deformed wing virus, cellular immunity genes are downregulated, whereas the expression of humoral immune genes increases [11]. Stress conditions, as induced by exposure to sublethal doses of neonicotinoids, also compromise the individual immunocompetence of honey bees [12].

Several AMPs, namely apidaecins, abaecin, defensins, and hymenoptaecin, were detected in the honey bee hemolymph [13,14,15,16,17]. AMPs are produced in fat bodies in bee abdomen and are released into the hemolymph [18]. AMPs gene expression can be triggered by a microbial challenge through the Toll, IMD-JNK, or JAK/STAT signalling pathways, but it has not yet been studied in bees, that were not stimulated by pathogen infection or in relation to bee nutritional status [5,19]. AMPs show a broad spectrum of antimicrobial or antifungal activities. On the one hand, defensin-1 is more effective towards Gram-positive bacteria; on the other hand, apidaecin isoforms and hymenoptaecin display higher activity against Gram-negative bacteria [15,20]. Focused on apidaecins, the analysis of cDNA displayed the occurrence of three coding sequences of apidaecins. The corresponding translated products are secreted as prepeptides: apidaecin type 73 (Q06602, UniProtKB), apidaecin type 22 (P35581, UniProtKB), apidaecin type 14 (Q06601, UniProtKB), coded by genes Apid73 (Gene ID 406115), Apid22 (Gene ID 494510), and Apid14 (Gene ID 406140), respectively. These prepeptides are finally spliced into active peptide isoforms: apidaecin 1A and 1B detected previously on the peptide levels, whereas apidaecin 2 has only been predicted from the cDNA library so far [13].

The published research work dealing with changes in the expression of genes for honey bee AMPs in response to specific stimuli has been mainly based on quantitative PCR analyses of bee tissues or whole bodies [21,22,23]. In addition, several other methods, such as polyacrylamide gel electrophoresis, high performance liquid chromatography, and mass spectrometry have been used for the detection and quantification of antimicrobial peptides in pooled samples of large numbers of bees [14,24,25]. Studies on bee AMPs concern mostly changes in the expression of the corresponding genes, as only a few reports on the quantification of AMP peptide levels have been published up to now. A new and highly sensitive analytical method has recently been developed for the quantification of apidaecin 1 isoforms in the hemolymph or body parts of individual bees, which is considered to be a promising tool for the accurate quantification of bee AMPs [26].

Here, we investigated changes in selected parameters of the humoral immune system in individual bees that were fed different pollen diets. We analysed the relative expression of genes coding for apidaecins and abaecin in honey bee abdomens, where they are synthetized in fat bodies. We also report the first quantitative measurement of levels of the active apidaecin 1 isoforms in bee thoraces, where they are present in circulating hemolymph, at the individual level of in vitro maintained bees.

## 2. Materials and Methods

### 2.1. Chemicals

Synthetic standards of apidaecin 1A and isotopically [^13^C_6_^15^N_4_] labelled apidaecin 1A (at the C-terminus) were synthetized by Clonestar (Brno, Czech Republic). Both synthetic peptide standards in solutions were quantified by the amino acid analysis by the Protein Analysis Group, Functional Genomics Centre, Swiss Federal Institute of Technology, Zürich, Switzerland. Acetonitrile, methanol, water (all of LC/MS grade quality), 98% (*v*/*v*) formic acid, and trifluoroacetic acid p.a. grade were from Sigma-Aldrich (Steinheim, Germany). 2-propanol for spectroscopy was from Merck (Darmstadt, Germany). All of the chemicals used in gene expression quantification were purchased in molecular biology quality.

### 2.2. Bee Rearing and Feeding

We incubated sealed brood combs from several colonies of *Apis mellifera carnica* from the Institute of Zoology, Graz, at 34.5 °C under standard conditions to obtain newly emerged honey bee workers, younger than 24 h [27]. Bees were chosen randomly and mixed before they were put into experimental cages consisting of clear plastic cups. Cages were also supplied with a wax bar and experiments were maintained for 18 days. Each cage contained 100 bees and the experiment was performed in duplicate cages per each type of the diet.

### 2.3. Preparation of Pollen Diets and Nutrition Factors

All of the bees were provided with 50% (*w*/*v*) sucrose solution *ad libitum*. All cages, except for one, were additionally fed one of the following protein diets: Feedbee^TM^, a supplementary protein diet available on the market, which does not contain any hive product [28], or corbicular pollen containing 94.8% of sunflower (*Helianthus* spp.), 91.2% of mustard (*Sinapis* spp.), 70.6% of asparagus (*Asparagus* spp.), or 87.6% of chestnut (*Castanea sativa*). See Omar et al. [29] for more details on diets. The monofloral pollen were collected by bees in Austria and kept frozen until use. The mixed pollen group received a 25% (*w*/*w*) mixture of each of the four different pollen-types. Each pollen load was palynologically analyzed at AGES—SPB, Abteilung, Bienenkunde und Bienenschutz, Lunz am See, Austria and kneaded into a dough that was provided to caged bees in one half of cylindrical 10 mL plastic tubes [27]. All of the bee diets were daily renewed and weighted. Mean cumulative consumption of diets during the 18 days of feeding ranged from 42.52 mg/bee for Asparagus to 68.28 mg/bee for the mixed pollen diet (see Supplementary Material S1).

### 2.4. Sample Pre-Treatment

Experimental bees were collected, immediately frozen, and stored at −80 °C until use. Before processing, individual frozen bees were dissected to whole thoraces, used for the quantification of apidaecin 1 isoforms, and abdomens, used for the quantification of gene expression of apidaecins and abaecin.

### 2.5. Quantification of Apidaecin 1 Isoforms

Bee thoraces were weighed without legs and wings before processing. Afterwards, they were homogenized with 600 µL of 0.1% *v*/*v* trifluoroacetic acid (TFA) using ceramic balls in 2-mL microtubes. The next steps were performed according to the protocol by Danihlík, Šebela, Petřivalský, and Lenobel [26]. Briefly, the homogenized bee thoraces were centrifuged, heated at 100 °C for 10 min to denature proteins, and centrifuged again. Then, aliquots (180 µL) of clarified supernatant were lyophilized and stored at −80 °C until further processing. Lyophilized samples were dissolved in 100 µL of 5% *v*/*v* formic acid (FA), and then 33 µL were loaded in technical duplicates onto WCX-Tip microcolumns filled with 5 mg of Oasis^®^ WCX 30 µm sorbent (Waters, Milford, MA, USA). Final enriched fractions of apidaecin 1 isoforms were evaporated and dissolved in 5% FA before a desalting step by reverse-phase liquid chromatography using C_8_-Stage Tips. Purified and desalted samples were diluted in 30 µL of 0.1% (*v*/*v*) TFA before nLC-MS analysis. The quantification of apidaecin 1 isoforms was performed on a nanoflow liquid chromatograph (RSLCnano) coupled via electrospray ion source with an ultra-high resolution Q-TOF mass spectrometer (UHR-Q-TOF maXis) controlled by CompassQTOF v 1.4 (Bruker Daltonics, Bremen, Germany). The concentration of apidaecins 1 in thoraces was calculated from a linear calibration curve that was constructed using a gradually increasing concentration of a synthetic external standard of apidaecin 1A mixed with a constant amount (1 pmol) of isotopically labelled [^13^C_6_^15^N_4_] apidaecin 1A, both diluted in a homogenate of freshly emerged bees purified using WCX-Tip microcolumns [26]. For detailed information about the set up of the nLC-MS method, see Supplementary Material S2.

### 2.6. Protein Assay

Bradford assay was applied in a microarray layout for protein quantification in samples. Bovine serum albumin served as a protein standard [30].

### 2.7. RNA Isolation and cDNA Preparation

Individual abdomens of bees were homogenized in the GITC buffer (300 µL per abdomen) [31]. The RNeasy Plant mini kit (Qiagen, Hilden, Germany) was used for RNA isolation. Homogenates (100 µL) were mixed with 350 µL of RTL buffer from the kit and processed, following manufacturer’s instructions. Finally, RNA concentration was quantified by absorbance at 260 nm using a BioSpec-nano micro-volume spectrophotometer (Shimadzu, Tokyo, Japan). Contaminating DNA was digested with Turbo DNAse (Ambion by Life Technologies, Carlsbad, CA, USA) (Supplementary Material S3). RNA integrity was checked by gel electrophoresis on 1.1% (*w*/*v*) agarose gel containing ethidium bromide. The Transcriptor High Fidelity kit (Roche, Basel, Switzerland) was used for cDNA synthesis from purified RNA, following manufacturer’s instructions. The quality of cDNA and possible presence of a genomic DNA contamination were tested by PCR (Supplementary Material S3) gel electrophoresis in 3% (*w*/*v*) agarose gel with ethidium bromide as a detection method and with a 50–1000 bp PCR Marker (Promega, Madison, WI, USA) as a standard.

### 2.8. Analysis of Gene Expression

Quantitative PCR (qPCR) reaction was performed on a CFX96 TouchTM Real-Time PCR Detection System (Bio Rad, Hercules, CA, USA) with SyberSelect^®^ Master Mix (Life Technologies, Carlsbad, CA, USA). The primer pair, which were designed to amplify the gene *Apid1* (synonymously *Apid14*-for primer sequences see Supplementary Material S3), is universal for all three existing apidaecin genes: *Apid14* (Gene ID 406140), *Apid22* (Gene ID 494510), and *Apid73* (Gene ID 406115). The gene expression was quantified in abdomens. The amplification efficiency was determined for all of the primers (Supplementary Material S4). The specificity of each qPCR gene expression assay was evaluated by the corresponding dissociation melting curve.

### 2.9. Quantification of Relative Gene Expression Level

The primers for the two housekeeping genes (*Arp1* and *EF1a-F2* were previously validated for various developmental stages of bee brood or tissues (brain, ovary, fat body, and hemocytes of queens) [32]. Here, these housekeeping genes (HKGs) were also validated for the whole bee abdomens. The stability of HKGs were calculated with BestKeeper© application, version 1 [33] (Supplementary Material S5).

Both selected HKGs, *Arp1* and *EF1a-F2*, were used for the normalization of the genes coding for apidaecins and abaecin peptides. qPCR data follows the MIQE guidelines [34] (Supplementary Material S6).

### 2.10. Statistical Analysis

The box plot graphs were constructed in OriginPro 9.0.0. The box plot graph shows 1st–3rd quartiles, whereas squares represent means and lines show medians. Basic statistics and parametric ANOVA or non-parametric Kruskal-Wallis and *post hoc* multiple mean or median comparison statistic tests were carried out in Statistica 13 (64-bit).

The expression stabilities of housekeeping genes (HKG) coding for *Arp1* (HKG1) and *EF1a-F2* (HKG2) were evaluated in BestKeeper [33], and the relative expressions of the genes of interest (GOI) for apidaecins (GOI1) and abaecin (GOI2) were calculated by comparing ratio between the expression of housekeeping genes and genes of interest corrected to their efficiencies [35,36].

## 3. Results

### 3.1. Effect of Pollen Diet on the Levels of Apidaecin 1 Isoforms in Bee Thorax

**Figure 1:**
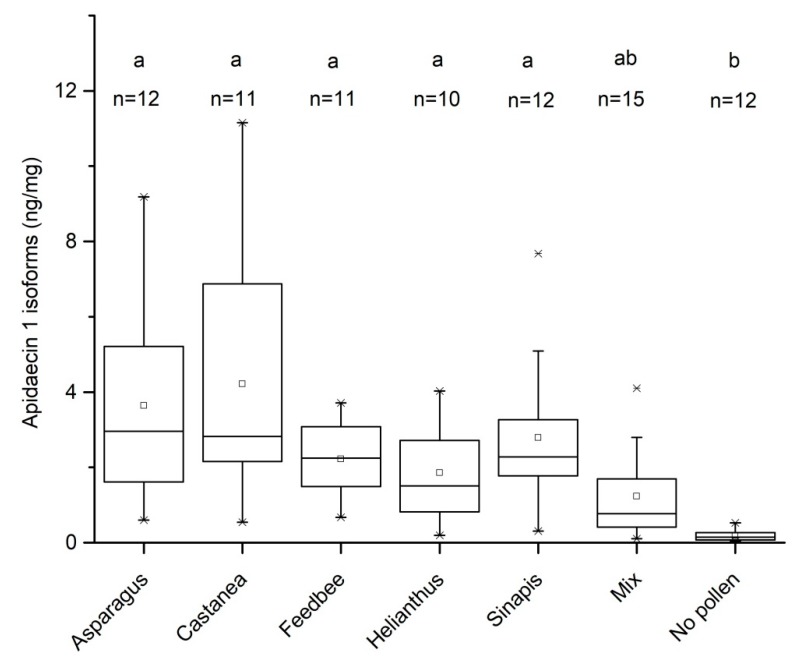
Concentration of apidaecin 1 isoforms in thoraces of caged bees fed different diets. Groups are labelled by small case letters; different letters denote significant differences between groups (independent-samples Kruskal-Wallis test, *p* < 0.05). Boxes show first and third interquartile range with line, median is denoted. Stars represent outliers, *n* = number of analysed individual bees, squares represent means.

When bees were fed no protein diet, the median amount of apidaecins 1 was 0.2 ng/mg, whereas all bees fed *Asparagus*, *Castanea*, and *Sinapis* pollen or FeeBee^TM^ diets during the first 18 days after their emergence showed higher concentrations (independent-samples Kruskal-Wallis test, *p* < 0.05; Figure 1). Mixed pollen diet resulted in the lower production of apidaecin 1 isoforms, but there was no significant difference when compared to no pollen fed group, as well as the other experimental groups.

There were no significant differences found in protein concentrations in homogenates of bee thoraces between bees that were fed non-pollen or any of the protein diets (one way Shapiro-Wilk normality test, *p* > 0.05; one way ANOVA, *p* > 0.05). Therefore, no pairwise comparisons were performed. The grand average of protein concentration was 65 µg protein per mg of homogenized thorax (95% CI: 60–70 µg/mg) (data not shown).

### 3.2. Effect of Pollen Diet on Expression of Apidaecin and Abaecin Genes

**Figure 2:**
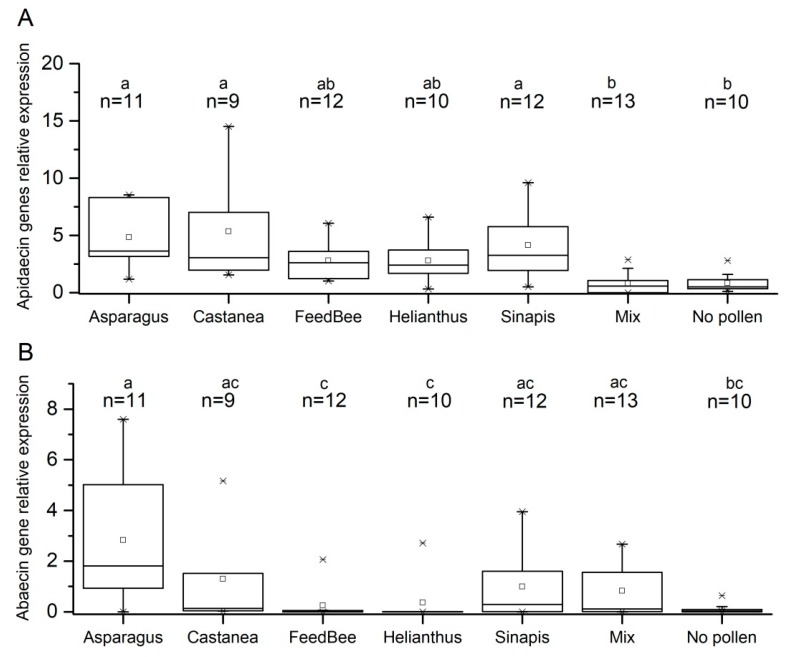
Relative expression of (**A**) apidaecin and (**B**) abaecin genes fed different diets. Groups are labelled by small case letters; different letters denote significant differences between groups (independent-samples Kruskal-Wallis test, *p* < 0.05). Boxes show first and third interquartile range with line, median is denoted. Stars represent outliers, *n* = number of analysed individual bees.

Different trends in apidaecin and abaecin gene expression were observed. As the normality of data distribution within experimental groups was rejected by Shapiro-Wilk test (*p* > 0.05), Kruskal-Wallis non parametric test and *post hoc* median comparison were used to evaluate the differences among experimental groups. Bees fed mixed pollen and no pollen showed significantly lower apidaecin gene expression when compared to bees fed *Asparagus*, *Sinapis,* or *Castanea* pollen (Figure 2A). No significant differences were observed among bees that were fed *Helianthus* pollen or FeedBee^TM^ when compared to a mixed pollen or no pollen diet.

The level of abaecin gene expression is generally lower as compared to apidaecin genes, as the overall experimental groups medians of relative gene expression for apidaecin and abaecin were found 3.00 and 0.950, respectively (Mann-Whitney U test, *p* < 0.001). Kruskal-Wallis test, followed by *post hoc* median comparisons showed unique significant difference in abaecin gene expression between bees that were fed *Asparagus* pollen and bees fed no pollen diet. Very low abaecin expressions were detected in bees fed *Helianthus* pollen and the pollen supplement FeedBee^TM^, but the difference was not significant to other groups.

### 3.3. Correlation of Apidaecin 1 Isoforms Concentration in Thoraces and Gene Expression in Abdomens

**Figure 3:**
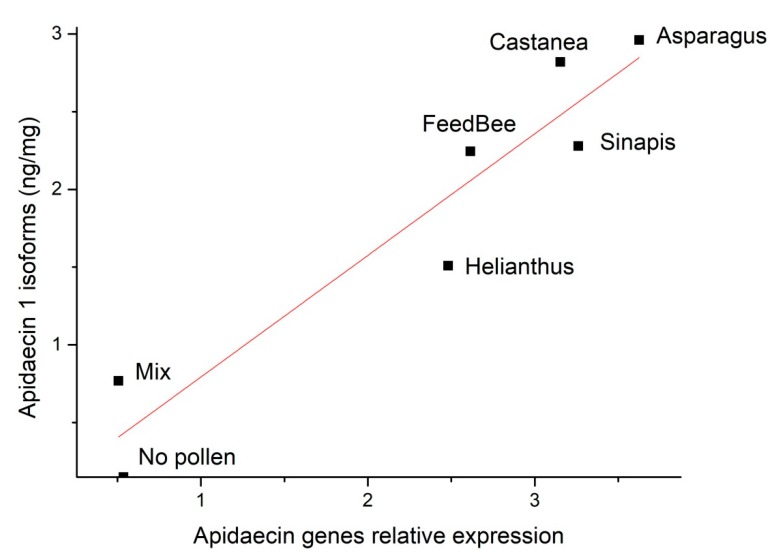
Correlation of apidaecin genes relative expression and apidaecin1 isoforms levels in bee groups that were fed different types of diet. Individual points represent median values of each group.

Medians of apidaecin concentrations in thoraces were plotted against medians of gene expression in abdomens of all of the experimental groups (Figure 3), and a simple linear regression model was established: y = 0.782_(SE = 0.11484)_x Pearson’s r = 0.95). A linear model with intercept was not accepted because the intercept was not significantly different from zero (95% CI = −0.76; 0.78).

## 4. Discussion

In general, the dietary protein is a prerequisite for protein synthesis, including the formation of the isoforms of the antimicrobial peptide apidaecin 1. All protein diets, except the mixed diet, increased the concentrations of apidaecin 1 isoforms in honey bee thoraces when compared to the no-pollen diet (Figure 1).

It is known that optimal protein nutrition should provide a balanced content of essential amino acids that is required for honey bees [37,38]. The quality of monofloral diets is dependent on how they fulfil this biochemical requirement. Some particular pollen, including that of *Helianthus*, can lack several amino acids, which are essential for honey bee nutrition [39]. On the other hand, chestnut pollen (*Castanea* spp.) is regarded to be of high nutritional value to honey bees [2]. Here, we show that bees that were fed *Castanea* pollen showed the highest increase in average apidaecin 1 isoforms levels in thoraces when compared to bees fed no pollen diet. In general, mixtures of different pollen diets are believed to be good nutrient sources for adult honey bees [2,29].

Bees randomly collected from colonies have been reported to contain 18–54 ng of apidaecin 1 in whole thoraces [26], which is in the same range as the bees fed pollen or FeedBee^TM^ diet in our experiment (1st–3rd quartile: 36.9–111.7 ng per thorax, recalculated data). Bees that were fed no protein in our study contained only 4.5 ng/thorax (median) (1st–3rd quartile: 2.3–8.3 ng per thorax), which is a significantly lower value when compared to bees that were collected from the hive [26]. In general, the difference between bees fed pollen or pollen supplement diet (*n* = 71) is significantly higher as compared to bees fed sugar syrup only (*n* = 12) (Mann-Whitney U test, *p* < 0.05).

A previous transcriptomic comparison of bees fed no pollen diet versus those fed pollen showed differences based on diet quality in gene expression in three or eight days old bees [40]. In our study, the pollen or FeedBee^TM^ supplemented diet resulted in significantly changed gene expression of antimicrobial peptides apidaecins and abaecin as compared to no protein diet (Figure 2). Our results suggest that a specific diet or its components of pollen origin can increase gene expression of antimicrobial peptides in non-infected bees. We found an upregulation of immunity related genes for all diets containing protein, which extends previous studies that found a similar increase in bees fed honey when compared to artificial carbohydrate syrups. It was identified that p-coumaric acid (a monomer of the principal constituent of pollen cell walls) and other substances that are usually found in propolis upregulate genes required for immune defence [41,42]. However, our findings show that a non-hive product protein like Feedbee^TM^ could also stimulate such a gene expression. Interestingly, increased gene expression of apidaecin was observed in bees that were fed *Asparagus* and *Castanea* pollen whereas abaecin gene expression was significantly increased by *Asparugus* pollen diet only. Similarly, in another study using the same pollen diets, caged bees fed *Asparagus* and *Castanea* pollen diet had largest hypopharyngeal glands and acid gland sacs [29].

Recent studies confirmed the changes in gene expression of AMPs within honey bee immune gene network in response to major bee pathogens and parasites [21,23,43,44]. However, it is widely known that the level of gene expression does not necessarily correlate with levels of active peptide molecules in bee tissue. This may occur e.g., when the peptide translation is regulated by microRNA [45,46]. Here, we found a positive correlation between apidaecin gene expression in the fat body and apidaecin levels in bee thoraces (Figure 3). Focused on apidaecins, they are secreted as preproteins: apidaecin type 73, type 22 and type 14, these preproteins are secreted by genes Apid73 (Gene ID 406115), Apid22 (Gene ID 494510), and Apid14 (Gene ID 406140). The preproteins are finally spliced into three active isoforms detected on peptide level: apidaecin 1 (isoforms 1A and 1B), apidaecin 2, and one predicted apidaecin from cDNA library [13,15].

Dietary proteins are necessary for the proper function of multiple immune pathways of honey bees [47]. Pollen diet, which is composed of pollen grains of many different botanical species, is often considered to be the most natural and nutritive diet for honey bees [48,49,50]. Previous studies on the suitability of artificial protein diets for honey bees brought controversial results depending on the investigated trait of honey bee vitality [50,51]. When compared to different pollen diets that were used in our experiment, no striking advantages or disadvantages of Feedbee^TM^ could be detected based on the evaluated parameters of innate immunity, i.e., apidaecin 1 isoforms concentration and apidaecins and abaecin genes expression. In our study, we could not unequivocally determine whether the observed up-regulation of the abaecin and apidaecins coding genes in bees fed pollen-supplemented diets was attributed only to the protein nutritional value of the diet or evoked by antigens or other chemical components. Together with other stress factors reducing immunocompetence of honey bees (e.g., pesticides), malnutrition may act synergistically influencing negatively bee immune pathways and the ability of defence against pathogens [52,53,54].

Besides the plant-derived pollen grain components, the importance of pollen-associated bacterial microbiota is poorly explored but an emerging field that is tightly related to the nutrition and health status of not only honey bees, but pollinator insects in general [55,56]. It was not the aim of our study to analyse the microbial flora of used experimental pollen diets. The pollen samples were not sterilized by gamma radiation or any other method which could influence the pollen quality, e.g., by destroying less stable compounds as vitamins, antioxidants, etc. Moreover, even the use of pollen sterilization to kill present microorganisms would not eliminate possible effects of dead bacterial cells or their components as potential triggers of bee immunity. In this respect, the relevance of both pathogenic and non-pathogenic microorganisms on the pollen surface to bee immunity tightly interconnected to the bee gut microbiome surely deserves further investigation. Indeed, evidence has been made that native non-pathogenic gut microbes induce AMP expression in the bee host, and that this might benefit bee health by priming the immune system against future infections [57]. However, we do not understand yet completely why bees that were fed different food sources modulate gene expression, and hence AMP concentrations are on high or low levels. Pollen alone could contain certain biomolecules that could activate immune reactions, what alternatively could explain the variable gene expression and production of antimicrobial peptides

## 5. Conclusions

We investigated the importance of protein nutrition for production of representatives of honey bee antimicrobial peptides apidaecins and abaecin. Our findings demonstrate the variable effects of protein (originating e.g., from a natural pollen diet) content and quality in adult honey bee nutrition to parameters of bee innate immunity, such as concentration of apidaecin 1 isoforms and the expression of genes for apidaecins and abaecin. Previous studies have mostly reported only the expression profiles of the genes coding for AMPs and not their peptide levels in bee tissues. We also found positive linear correlation between apidaecin 1 isoforms concentration and expression of genes for apidaecins. This work shows that analysing the levels of active AMPs in individual bees, in addition to analysis of pooled samples, may reveal interesting findings that are related to bee physiology. The results of our study confirmed that the apidaecin 1 isoforms concentration are significantly influenced by pollen dietary protein. Our findings on the importance of protein content in bee diet suggest wide practical implications in reconsidering the importance of protein supplementation to sugar-based diet provided to bee colonies and its impact on the bee and colony health status and the capacity of successful overwintering. Further experiments are needed to dissect which pollen diet components of either plant or microbial origin have key roles in the activation of bee innate immunity, namely in conditions of bees that are exposed to pathogenic challenge.

## Supplementary Materials

The following are available online at http://www.mdpi.com/2075-4450/9/3/79/s1. Supplementary data S1: diet consumption; Supplementary data S2: nLC-MS method details; Supplementary data S3: sample preparation for gene expression; Supplementary data S4: primer efficiency; Supplementary data S5: validation of reference genes; Supplementary data S6: MIQE table.

## Abbreviations

AMP	antimicrobial peptide
Cq	quantification cycle
CI	Confidence interval
FA	formic acid
GITC	guanidium isothiocyanate
GOI	gene of interest
HKG	housekeeping gene
MALDI-TOF MS	matrix-assisted laser desorption/ionisation time-of-flight mass spectrometry
nLC-MS	nanoflow liquid chromatography coupled with mass spectrometry
qPCR	quantitive polymerase chain reaction
RP-HPLC	reversed-phase high performance liquid chromatography
TFA	trifluoroacetic acid
UHR-Q-TOF	ultra-high resolution quadrupole-time-of-flight
WCX	weak cation exchange
