# Supplementary material for: Does the Pollen Diet Influence the Production and Expression of Antimicrobial Peptides in Individual Honey Bees?
Source: Insects. 2018 Jul 4;9(3):79. doi: 10.3390/insects9030079 (PMC6164669; doi:10.3390/insects9030079)
Supplement: Supplementary file 1 [file insects-09-00079-s001.zip › Supplementary Material S1-S5.pdf]

insects

Jiří Danihlík, Mária Škrabišová, René Lenobel, Marek Šebela, Eslam Omar, Marek Petřivalský, Karl Crailsheim, Robert Brodschneider (2018)

Does the pollen diet influence the production and expression of antimicrobial peptides in individual honey bees?

Jiří Danihlík [1,2], Mária Škrabišová [3], René Lenobel [1], Marek Šebela [1], Eslam Omar [4], Marek Petřivalský [2], Karl Crailsheim [4] and Robert Brodschneider [4,*]

Supplementary data S1:

Table S1. Cumulative diet consumption (mg per bee) during the 18 days feeding for replicate 1 (R1) and replicate 2 (R2) and mean.

	R1	R2	Mean
**Feedbee®**	62,76	64,88	63,82
**Helianthus sp.**	55,22	60,17	57,7
**Sinapis sp.**	45,41	51,62	48,52
**Asparagus sp.**	43,87	41,16	42,52
**Castanea sp.**	58,86	60,76	59,81
**Mix pollen**	69,56	66,99	68,28

Supplementary data S2:

Nanoflow liquid chromatography coupled with mass spectrometry (nLC-MS)

Apidaecin 1 isoforms were identified and quantified according to procedure by Danihlík, J., Šebela, M., Petřivalský, M., Lenobel, R., 2014. A sensitive quantification of the peptide apidaecin 1 isoforms in single bee tissues using a weak cation exchange pre-separation and nanocapillary liquid chromatography coupled with mass spectrometry. Journal of Chromatography A 1374, 134-144 with minor modifications.

The coupled system used for the identification and quantification of apidaecin 1 isoforms consisted of a nanoflow liquid capillary chromatograph (Ultimate 3000 RSLCnano, Thermo Fisher Scientific, USA) connected to an ultra-high resolution Q-TOF mass spectrometer (UHR-Q-TOF maXis, Bruker Daltonik, Bremen, Germany) via a nano-electrospray ion source Captive Spray(Bruker Daltonik). The system was controlled by CompassQTOF v 1.4 and HyStar v3.2 software (Bruker Daltonik). Loading and separation of samples was carried out in a one-column setup (i. e. without any precolumn). There was an analytical column (75 µm i. d. × 100 mm) ended with an IntegraFrit (New Objective, Inc., MA, USA) and packed with a C_4 sorbent (ReproSil-Gold C_4, 300Å, 5 µm, Dr. Maisch; Ammerbuch-Entringen, Germany) in length of 5 cm. The composition of mobile phase A was 0.4% (v/v) formic acid (FA); mobile phase B consisted of 0.4% (v/v) FA in 90% (v/v) acetonitrile. Dried samples were dissolved in 30 µL of 0.1% trifluoroacetic acid (TFA), vortexed, sonicated for 5 min and transferred into total recovery glass vials (Waters, USA). An aliquot of the sample (5 µl) was loaded and washed on the column using a loading mobile phase (2% v/v FA) at a flow rate of 3 µl/min for 7 min. The retained peptides were eluted with a 25-min-long gradient (all mobile phase changes were linear: 0 min, 2% B; 3 min, 8% B; 15 min, 65% B; 16 min, 85% B; 21 min, 85% B; 22 min, 2% B; 25 min, 2% B) at a flow rate of 300 nl/min. Each sample was injected twice to check the reproducibility of response during the process of electrospray ionization.

insects MDPI

Full MS spectra were acquired in the positive ionization mode in the range of *m/z* 350 – 1600 with a frequency of 0.5 s per mass spectrum. Prior to analysis, the mass spectrometer was always calibrated in the *m/z* range of 118-2722 (ESI-L TuneMix, Agilent Technologies Part. No: G1969-85000) and tuned for a minimal resolution of 25,000 full width at half maximum (FWHM) using a calibration ion at *m/z* 922.0098. Source settings included capillary voltage of 1200 V, dry gas flow rate of 4 l/min, and dry temperature of 130 °C. All raw data were processed using DataAnalysis software v 4.0 SP5 (Bruker Daltonik). Targeted analytes (apidaecin 1 isoforms and the internal standard $[_{13}C^{6}{}_{15}N^{4}]$apidaecin 1A) were checked in a defined retention window of ± 0.5 min. Data analysis for analytes were carried out by extracting high-resolution accurate mass traces (± 0.005 Da) concerning the most dominating protonated ion charge state.

External calibration

For quantification of the level of apidaecin 1 isoforms in thorax samples, a calibration curve was generated using the standards of apidaecin 1A and $[_{13}C^{6}{}_{15}N^{4}]$apidaecin 1A diluted in a purified homogenate from freshly emerged bees: apidaecin 1A was mixed in a concentration range of 0 to 5 pmol with a fixed amount (1 pmol) of $[_{13}C^{6}{}_{15}N^{4}]$apidaecin 1A in 30 µl of 0.1% (v/v) TFA. High-resolution extracted ion chromatograms (HR-EIC) were generated as a sum of the two most intense quadruply charged abundant isotopic peaks ($[M+4H]^{4+}$) ions of apidaecin 1A at *m/z* 527.80796 plus 528.05048 and of $[_{13}C^{6}{}_{15}N^{4}]$apidaecin 1A at *m/z* 530.30298 plus 530.55549. Areas under extracted ion chromatographic peaks were integrated to get total peak areas. Then the ratios of the peak areas of apidaecin 1A *vs* $[_{13}C^{6}{}_{15}N^{4}]$apidaecin 1A were plotted against spiked amounts of apidaecin 1A (in pmol).

Quantification of apidaecin 1 isoforms in bee thoraces

Apidaecin 1 isoforms were isolated by the protocol based on weak cation exchange chromatography microcolumns (5 mg of Oasis® WCX 30 µm sorbent, Waters, USA) and analysed by nLC-MS. Collected raw data were processed in the DataAnalysis v 4.0 SP5 software as described above and the ratio of the peak areas of apidaecins 1 vs $[_{13}C^{6}{}_{15}N^{4}]$apidaecin 1A was calculated. The content of apidaecin 1 isoforms in samples was estimated from the calibration curve and recalculated to the original thorax samples. The diluting factor for the content of apidaecin 1 isoforms in homogenates and hemolymph was 10. Only 180 µl were used for lyophilization from the total homogenate volume of 600 µl. Then the dried sample was reconstituted in 100 µl of 5% FA from which a 33-µl aliquot was loaded onto the WCX-Tip microcolumn. Apidaecin 1 isoforms level in the thorax homogenates was calculated according to the following equation: apidaecins 1 (ng) = determined quantity (pmol) × 10 (diluting factor) × 2107 (molecular weight of apidaecin 1) × 0.001.

Supplementary data S3:

Contaminating DNA digestion

The sample was incubated with 2U of the enzyme at 37 °C for 30 min. This procedure was repeated two times. Then the RNA was immediately purified using paramagnetic beads Agercourt RNAclean XP (Beckman Coulter) with a slight modification of manufacturer’s protocol. Briefly, 50 µl of magnetic particles and 40 µl of isopropanol were mixed with approximately 100 µl of an RNA sample. The mixture was incubated at laboratory temperature for 10 min and washed 3 times with 70% (v/v) ethanol. Finally, the RNA was eluted by 32 µl of RNase-free water.

cDNA synthesis and endpoint PCR

insects

Set up for PCR reaction: 4.75 µl of water, 0.5 µl of 10 µM forward primer, 0.5 µl of 10 µM reverse primer, 0.5 µL of template and 6.25 µl of GoTaq® Green Master Mix 2× (Promega). For the composition of primers, see Table S2. PCR cycling was programmed as follows: 95 °C - 2 min, 35×[95 °C - 40 s, 60 °C - 30 s, 72 °C - min], 72 °C - 5 min, 4 °C.

Quantitative PCR

Quantitative PCR (qPCR) reaction was performed in a total reaction volume of 5 µL: 2.5 µl of 2× SyberSelect® Master Mix, 0.45 µl of 3.3 µM of forward primer, 0.45 µl of 3.3 µM reverse primer and 1.6 µl of the template; templates were used in a concentration range recommended by the manufacturer. The qPCR reaction set up was as follows: denaturation at 95 °C for 10 min, 40 × [95 °C - 15 s, 60 °C for 1 min], followed by a melting curve analysis where the dissociation curve was assessed for product size confirmation. The same primers were used as in the above PCR (Table S2).

Table S2. Primer sequences and the corresponding target gene names used for PCR reactions.

Peptid/protein	Gene name	Gene ID	Amplicon size (bp)	Primer 5′→ 3′		T_m (°C)	Reference
Apidaecin type 14	*Apid1*	406140	80	F	TTTTGCCTTAGCAATTCTTGTTG	60.0	(Simone et al., 2009)
				R	GTAGGTCGAGTAGGCGGATCT	63.4	
Actin related protein	*Arp1*	406122	155	F	TGCCAACACTGTCCTTTCTG	64.0	(Lourenço et al., 2008)
				R	AGAATTGACCCACCAATCCA	64.1	
EF-1a	*EF1a-F2*	544670	153	F	GGAGATGCTGCCATCGTTAT	63.9	(Lourenço et al., 2008)
				R	CAGCAGCGTCCTTGAAAGTT	64.4	

Supplementary data S4:

The cDNA was geometricaly diluted with a factor 5.

Primer efficiency of gene coding for apidaecins was determined 111.64 %.

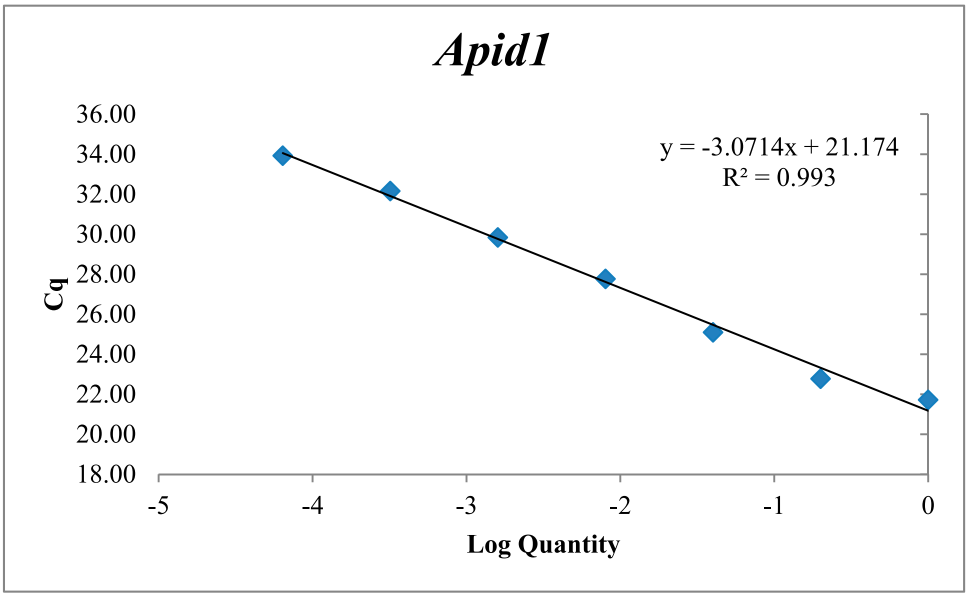

Primer efficiency of gene coding for actin was determined 100.87 %.

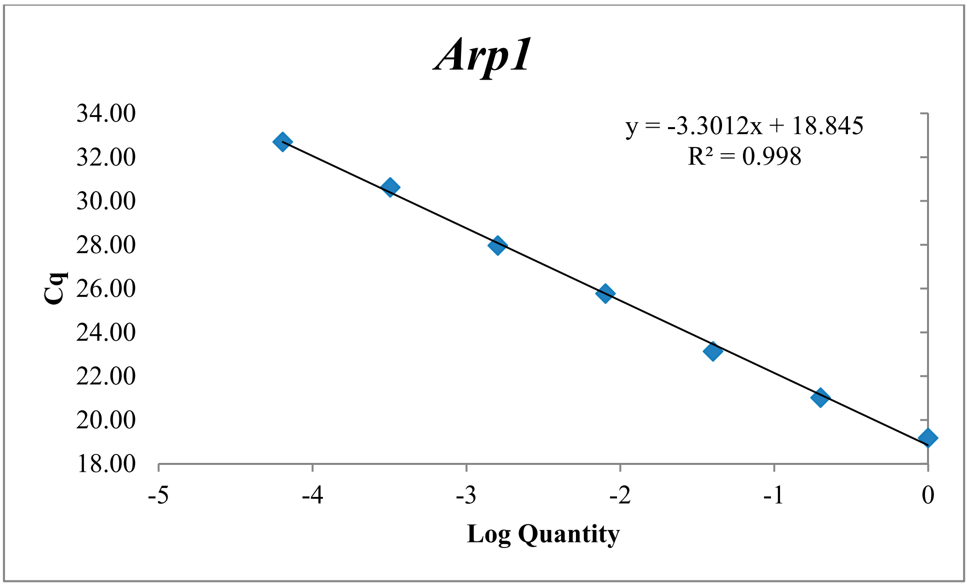

Primer efficiency of gene coding for EF1-α was determined 101.80 %.

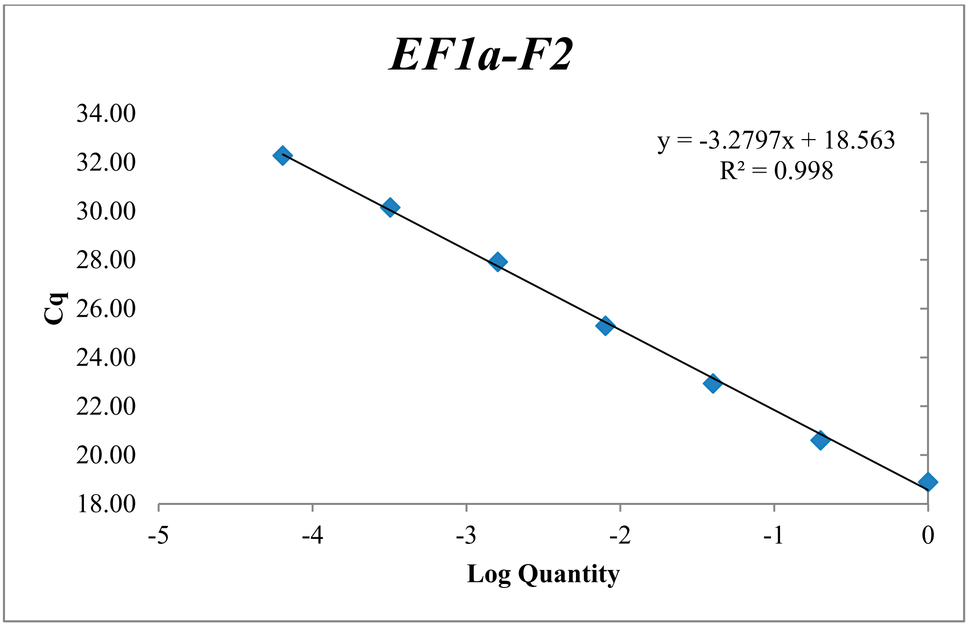

insects

Primer efficiency of gene coding for abaecin was determined 94.49 %.

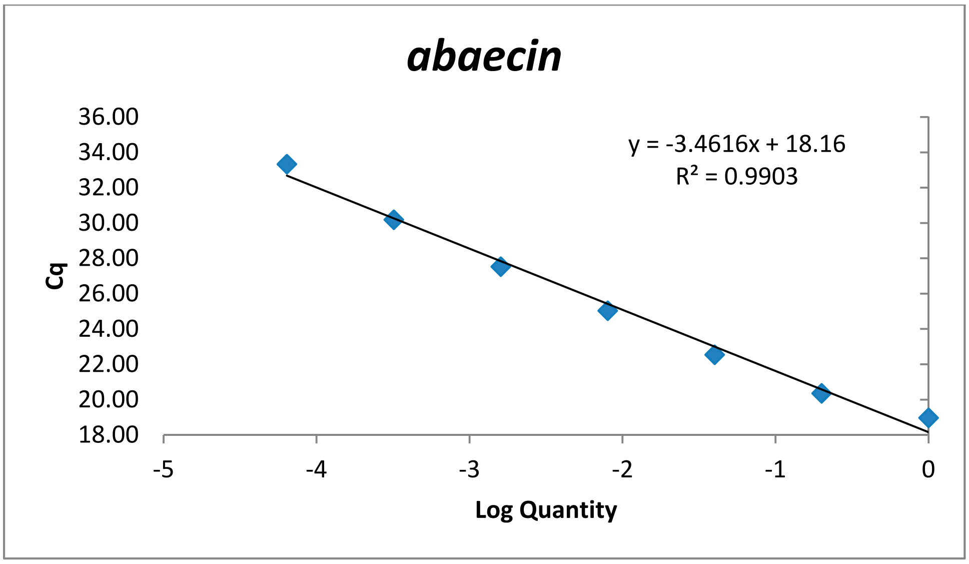

Supplementary data S5:

Validation of reference genes

Regression analysis of housekeeping genes vs. BestKeeper© application for Excel (Pfaffl et al., 2004). The presented results are from 81 PCR reactions from 81 samples of honey bee abdomens.

	EF1a-F2 housekeeping gene 1 vs. BestKeeper©	*Arp1* housekeeping gene 2 vs. BestKeeper©
coeff. of corr. [R]	0.917	0.937
coeff. of det. [R²]	0.841	0.878
intercept [Cq]	1.4881	-1.4772
slope [Cq]	0.9205	1.0792
SE [Cq]	±0.301	±0.303
p-value	0.001	0.001
Power of hosekeeping gene [x-fold]	1.908446	2.123127

Two reference genes, *Arp1* and *EF1a-F2*, were utilized for the normalization and calculation of the relative gene expression level for all samples. These x-fold expression results for RGs were corrected using the determined PCR efficiency. Neither *Arp1* nor *EF1a-F2* showed minimum or maximum x-fold values higher than 3. This limit is used for integrity analysis of a single sample. Based on this, both analyzed genes could be considered suitable RGs.
